# Pituitary adenoma with gangliocytoma: Report of two cases

**DOI:** 10.3892/ol.2014.2183

**Published:** 2014-05-27

**Authors:** DANQI CHEN, JIAN XU, PING ZHONG, XIANG HUANG, MING XU

**Affiliations:** Department of Neurosurgery, Huashan Hospital, Fudan University, Shanghai 200040, P.R. China

**Keywords:** gangliocytoma, pituitary adenoma, sellar region

## Abstract

Worldwide, cases of pituitary adenoma with gangliocytoma are rarely reported. The current study reports the cases of two 47-year-old females who presented with masses in the sellar region following a general examination and radiological imaging. The two patients underwent sellar region tumor resection via the trans-naso-sphenoid approach. The histopathological examination confirmed the diagnosis of a hormone-free pituitary adenoma with gangliocytoma. The two patients were in good condition and experienced no specific discomfort subsequent to the follow-up after surgery. Gangliocytoma is a slowly growing and non-metastasizing tumor. A biopsy is required to differentiate a gangliocytoma from a malignant neuroblastoma, and excision is usually curative.

## Introduction

Pituitary adenoma is the most common type of tumor found in the sellar region. It is believed to account for 10–15% of all primary brain tumors (1). Gangliocytomas [World Health Organization (WHO) grade I) are rare benign tumors of the sympathetic nerve fibers arising from neural crest cells, which can grow wherever sympathetic nervous tissue is found (1). Gangliocytoma commonly occurs in adolescents and young adults (40–60%), but individuals of all ages may be affected. Development of this tumor occurs at the extracranial and intracranial sites, with an incidence rate of ~0.5% at the intracranial site (2). The current study presents two cases of a hormone-free pituitary adenoma with gangliocytoma in the sellar region.

## Case reports

### Case one

A 47-year-old female presented to the Huashan Hospital (Fudan University, Shanghai, China) with blurred vision that had been present for the past two years. The patient had no obvious symptoms of headaches, nausea or vomiting, no notable polydipsia or diuresis and no phantom pain, limb activity disorder or body convulsions. However, two years prior to admittance, the patient was diagnosed with amenorrhea. One year prior to admittance, magnetic resonance imaging (MRI) revealed a lesion in the sellar region.

Upon neurological examination, a diagnosis of acromegaly was formed. The patient was able to fix and follow objects with each eye. Light perception was only present in the right eye, and the vision in the left eye was 0.2 decimal units. The fundus was unremarkable and the pupils were regular and reactive.

**Figure 1:**
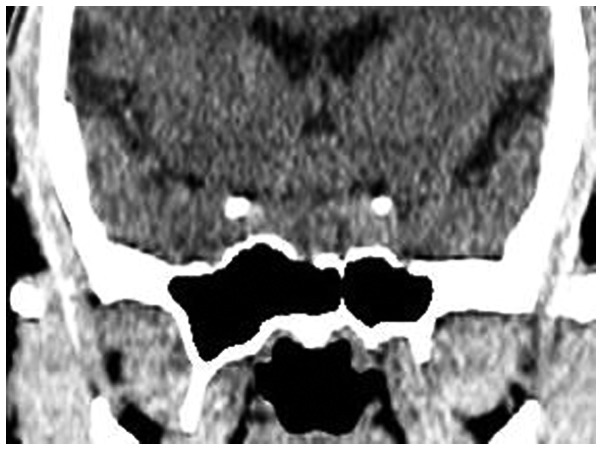
Computed tomography (CT) showing a parenchymatous mass in the sellar region, damage of the sclerotin of the anterior clinoid process, partial obliteration of the suprasellar cistern and a pressurized anterior third ventricle and precornu.

**Figure 2:**
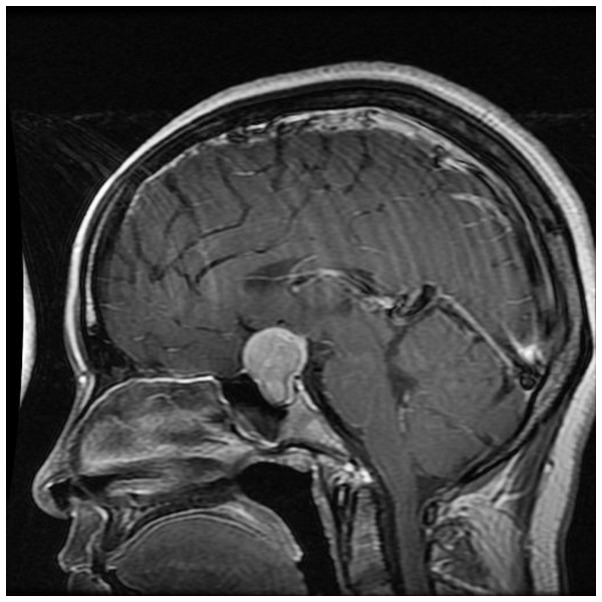
MRI showing a sellar lesion, and heterogeneous enhancement on contrast MRI. MRI, magnetic resonance imaging.

A computed tomography (CT) scan (Fig. 1) revealed a parenchymatous mass in the sellar region, damage of the sclerotin of the anterior clinoid process, partial obliterations of the suprasellar cistern and a compressed anterior third ventricle and precornu. MRI (Fig. 2) showed a sellar lesion, with heterogeneous enhancement on the contrast MRI.

A perimetry examination showed complete defects of the right eye in the 30 degree field, and defects of the left eye on the side of the temporal bone.

Pre-operative laboratory examinations found 1.21 mmol/l triiodothyrine (T3), 3.31 pmol/l free T3 (FT3), 10.28 pmol/l free thyroxine (FT4), 64.7 pg/ml adrenocorticotropic hormone (ACTH) and 8.9 mU/l growth hormone (GH). Post-operative laboratory examinations found <0.84 mmol/l TT3, 2.42pmol/l FT3, 9.36 pmol/l FT4, 19.0 pg/ml ACTH and 28.7 mU/l GH.

The patient was hospitalized due to a space-occupying lesion found in the sellar region in January 2012, and underwent sellar region tumor resection via the trans-naso-sphenoid approach. During surgery, it was found that the tumor exhibited a gray-red color and a heterogeneous texture. Softly-textured tumor sections were excised and examined under microscope, together with firmly-textured tumor sections, which were hard to excise.

**Figure 3:**
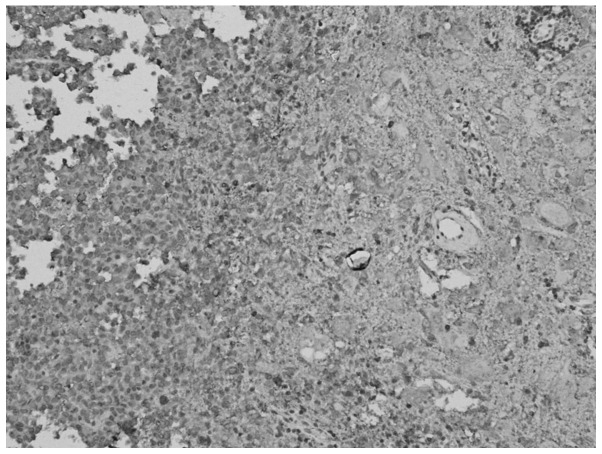
Two types of tumor components found under spectroscopy. Certain areas show a nest-shaped distribution of the cell debris of the pituitary adenoma. Other areas show a laminar distribution of ganglion cell-like neurons.

Post-operative pathological examination confirmed the diagnosis of a hormone-free pituitary adenoma with gangliocytoma [World Health Organization (WHO) grade I]. Under a spectroscope, (Fig. 3) two types of tumor components were found; certain areas showed a nest-shaped distribution of the cell debris of the pituitary adenoma, and were positively stained for Syn and negatively stained for GH, luteinizing hormone (LH), prolactin (PRL), thyroid-stimulating hormone (TSH), follicle-stimulating hormone (FSH) and ACTH. Other areas showed a laminar distribution of ganglion cell-like neurons and were positively stained for Syn and negatively stained for cluster of differentiation 34. MIB-1 expression was <1%.

Subsequent to a three-month follow-up period, the patient’s vision in the left eye remained at 0.2, while the vision in the right eye increased to 0.1.

### Case two

A 47-year-old female presented to the Huashan Hospital with a lesion in the sellar region, which was found one month previously on MRI examination due to a head trauma. The patient typically had good vision and in recent days had no notable polydipsia or diuresis and no phantom pain, limb activity disorder or body convulsions. However, one year prior to admittance, the patient was diagnosed with amenorrhea.

Upon neurological examination, the patient was able to fix and follow objects with each eye, and the vision of each eye was 0.8 decimal units. The fundus was unremarkable and the pupils were regular and reactive.

**Figure 4:**
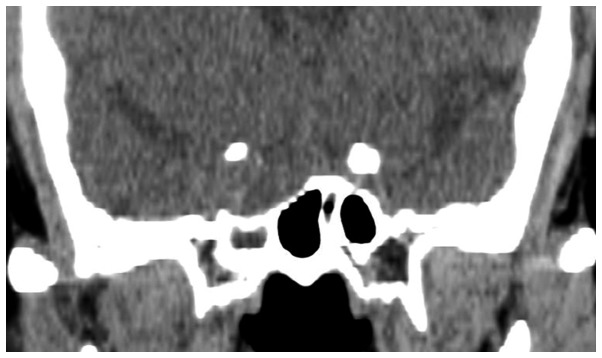
Computed tomography (CT) showing a space-occupying lesion in the sellar area, which affected the right-hand side. The sellar area is expanded and the end of the saddle has sunk.

**Figure 5:**
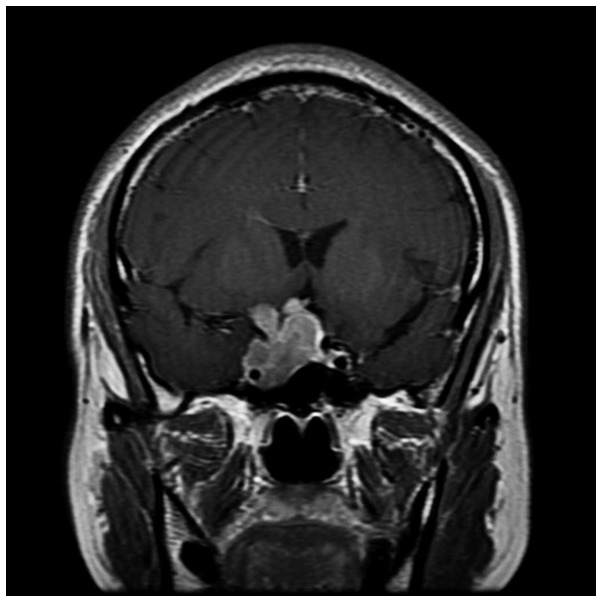
MRI showing an irregular abnormal signal of ~3.7×2.1 cm in size, with marked heterogeneous enhancement on the contrast MRI, which affected the right cavernous sinus and surrounded the right internal carotid artery. The pituitary stalk was compressed to the left, which caused obliterations of suprasellar cistern. MRI, magnetic resonance imaging.

A CT scan (Fig. 4) showed that there was a space-occupying lesion in the sellar area, which was situated on the right-hand side. The sellar area was expanded and the end of the saddle had sunk. MRI (Fig. 5) revealed an irregular abnormal signal measuring ~3.7×2.1 cm in size and marked heterogeneous enhancement on the contrast MRI, which affected the right cavernous sinus and surrounded the right internal carotid artery. The pituitary stalk was compressed to the left, which caused obliterations of the suprasellar cistern.

Perimetry and endocrinological examinations of the eyes were normal.

The patient was hospitalized due to the presence of a space-occupying lesion of the sellar region in March 2012, and underwent a sellar region tumor resection via the trans-naso-sphenoid approach. During surgery, it was found that the tumor exhibited a gray-red color, a firm texture and an adequate blood supply. The majority of the tumor was excised under a microscope. Post-operative pathological examination confirmed the diagnosis of a hormone-free pituitary adenoma with gangliocytoma (WHO grade I).

**Figure 6:**
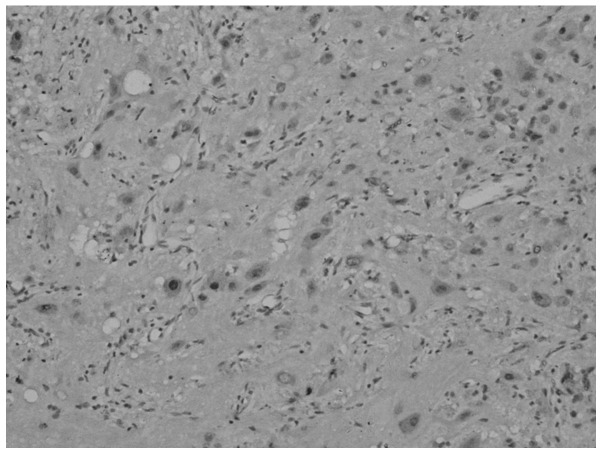
Two types of tumor components found under spectroscopy. Certain areas show a nest-shaped distribution of the cell debris of the pituitary adenoma. Other areas show a laminar distribution of ganglion cell-like neurons, with collagenous fibrosis and mild calcification.

Under a spectroscope (Fig. 6), two types of tumor components were found; certain areas of the tumor displayed a nest-shaped distribution of the cell debris of the pituitary adenoma, and other areas showed a laminar distribution of ganglion cell-like neurons with collagenous fibrosis and mild calcification. Immunohistochemical analysis of the cells of the pituitary adenoma revealed the following: Positive staining for Syn, and negative staining for GH, LH, PRL, TSH, FSH and ACTH. MIB-1 expression was <1%. The ganglion cell-like neurons were positive for Syn and NeuN, and MIB-1 expression was <1%.

Subsequent to a one-month follow-up period, the patient’s vision was restored to the same quality as prior to the surgery, with no significant changes observed. The patient was in a good condition and experienced no specific discomfort.

## Discussion

Pituitary adenoma is a common benign tumor of the sellar region, which typically originates from the anterior pituitary, while gangliocytoma is a rare benign tumor of neuroblastic origin, which originates from the posterior pituitary (1). However, these mixed pituitary tumors have been rarely reported worldwide.

Gangliocytoma (WHO grade I) originates from the neuron, and is attributable to neuronal and mixed neuronal-glial tumors. Neuroblastic tumors can be broadly subcategorized as neuroblastoma, ganglioneuroblastoma or gangliocytoma (2). The three tumors differ in their degree of cellular and extracellular maturation. The most benign tumor is the gangliocytoma, which is composed entirely of neural elements, including mature ganglion cells and schwannian stroma, and does not contain neuroblasts, intermediate cells or mitotic figures (3).

Gangliocytoma commonly occurs in adolescents and young adults (40–60%), but individuals of all ages can be affected. Development of this tumor is common at a young age and occurs at the extracranial and intracranial sites, with an incidence rate of ~0.5% at the intracranial site (3). Gangliocytoma in the intracranial site is prone to be distributed in the end of the third ventricle and in the frontal and temporal lobes (4), and is rarely found in the sellar region. Greenfield (5) reported one type of tumor that originated from the anterior and posterior pituitary of the sellar region in 1919, which was originally named a choristoma. Towfighi *et al* (6) reported 42 cases of ganglion cell tumors in the posterior pituitary in 1996; of these, 32 cases were mixed pituitary adenomas with gangliogliomas. Lu and Xu (7) did not find any cases of gangliocytoma through the pathological analysis of 1,458 cases of sellar region tumors. Qin and Yan (8) reported a 39-year-old male patient with gangliocytoma in the sellar region in 2004.

Gangliocytomas in the sellar region should be distinguished from pituitary adenomas. A total of 65% of pituitary gangliocytomas are accompanied with pituitary adenomas, and there are no significant differences in their clinical findings. As gangliocytomas mainly occur in the posterior pituitary lobe, patients may complain of endocrine symptoms mainly due to acromegaly and lactation menopause syndrome (2). In the two cases reported in the present study, the patients had previously been diagnosed with amenorrhea. One patient complained of acromegaly and their GH level was marginally elevated.

MRI and CT scanning are the preferred methods for imaging gangliocytomas (9). Non-enhanced CT scanning reveals a homogeneous mass with less attenuation compared with muscle. CT may show calcifications in two-thirds of cases. Calcification is typically fine and speckled, but may also be coarse (10,11). MRI is the modality of choice for evaluating the extension of the lesion. Gangliocytomas appear homogeneous on MRI and have relatively intermediate signal intensity on all pulse sequences. The density, signal and contrast manifestations are similar to those of a pituitary adenoma, therefore, it is difficult to distinguish them under MRI and other imaging modalities prior to surgery (8).

Gangliocytoma is a slowly growing and non-metastasizing tumor. A biopsy is required to differentiate gangliocytomas from malignant neuroblastomas, and excision is usually curative. During the surgery of the present study, it was found that the texture of the gangliocytoma was significantly more rigid and tougher than that of the pituitary tumor section, and the blood supply of the gangliocytoma was moderate. Gangliocytomas, as fully differentiated neoplasms, do not have the capability to metastasize, thus extensive surgical resections or chemotherapy are not typically required (12). The therapeutic effects are satisfied by surgery alone; recurrence is predominantly caused by the subtotal resection of the tumor and any clinical symptoms can be markedly improved by repeating the surgery.

In the present study, the gross pathology of the gangliocytoma specimens showed that the tumor had a firm texture with clear borders, and certain sections were accompanied by cystic lesions and calcification.

Spectroscopic examinations can reveal that under the eosinophilic glial background, differently sized and shaped gangliocytes exhibit irregular distribution patterns (mononuclear, dinuclear or polynuclear) and Nissl bodies in the cytoplasm. The tumor tissues contain nerve fibers with and without medullary sheaths, and immunohistochemical analysis reveals positive staining for NF, NSE, Syn and chromogranin A (13).

Gangliocytomas must be differentiated from hamartoma (14). Microscopically, a hamartoma is shown as ectopic nervous tissue of the pituitary, and is combined with ganglion cells, astrocytes and branch cells to form nodular prominents. Histologically, it is not a tumor, but the accrementition of ganglion cells, astrocytes and branch cells, which have been differentiated maturely. Gangliocytomas also require differentiation from the normal neurohypophysis, which is the normal tissue changing, in which spindle-shaped glial cells are sparse, and there are no metatypical cells, no hemorrhage and necrosis, and no Rosenthol fibers and eosinophilic bodies.

Geddes (15) *et al* hypothesized that the GH-releasing hormone (GHRH) produced by ganglion cells, which were heterotopic in the intrasellar region, may stimulate or accelerate the occurrence of the endocrine activity of the pituitary adenoma. Another possibility is that the undifferentiated ganglion cells discarded by the neurohypophysis transformed into tumor cells due to GHRH, which was excessively produced by the hypothalamus, while the pituitary cells formed the pituitary adenoma by GHRH stimulation. Another previous study has argued that the increasing number of hormone-releasing factors discharged by the hypothalamic neuron growing in the pituitary stimulate the proliferation of pituitary endocrine cells, while the ganglion cells form the mixed pituitary tumors, which are stimulated by the hormone produced by the pituitary (16). Ulm *et al* (17,18) argued that the mixed pituitary tumor originates from the steroidogenic acute regulatory protein-expressing follicular cells in the anterior pituitary, which are the primitive, pluripotent stem cells of the adult pituitary, with the capability of bidirectional differentiation of the adenohypophysis and neurohypophysis, and thus form tumors.

In summary, the possible genesis theories of the miscellaneous tumor include the following: i) The stem cell differentiation theory; ii) the induced theory, a tumor occurs first and induces another type of tumor occurrence; iii) the encounter theory, two tumors of different origins occur respectively and mix together; iv) the metaplasia theory, one tumor is the primary tumor, while the other is the metaplastic tumor; and v) the gene theory, tumorigenic factors act on the oncogenes of the adenohypophysis and neurohypophysis, and activate cell tumorigenesis synchronously or successively (17,18).

These mixed pituitary tumors have rarely been reported worldwide and therefore, future surgeries are required to confirm the pathological diagnosis and that generally, excision is curative. Since mixed tumors do not have the ability to metastatsize, the therapeutic effects may be satisfied by surgery alone. Recurrence is predominantly caused by the subtotal resection of the tumor and any clinical symptoms may be improved a second surgery. Therefore, this report may aid neurological surgeons’ understanding of mixed pituitary tumors.
